# Phage-antibiotic synergistic effect for treating cutaneous wounds infections caused by MRSA and the assessment of wound healing biomarkers in a rabbit model

**DOI:** 10.1038/s41598-025-34474-6

**Published:** 2026-02-12

**Authors:** Safia Samir, Hend Okasha, Tarek Aboushousha, Abdul Rahman Abu Seada, Sami Mohamed Nasr

**Affiliations:** 1 Biochemistry and Molecular Biology Department, Theodor Bilharz Research Institute (TBRI), Giza, Egypt; 2 Department of Pathology, Theodor Bilharz Research Institute (TBRI), Giza, Egypt; 3 Department of Anaesthesia and Surgical Intensive Care, Theodor Bilharz Research Institute (TBRI), Giza, Egypt; 4 School of Biotechnology, Badr University in Cairo, Badr City, Cairo 11829 Egypt

**Keywords:** MRSA, Wound healing, Wound infection, Antibacterial activity, Prophages, In vivo rabbit model, Biotechnology, Diseases, Microbiology

## Abstract

Antibiotic-resistant methicillin-resistant *Staphylococcus aureus* (MRSA) poses a significant challenge in managing wound infections. Bacteriophage therapy has emerged as a promising alternative or adjunct to traditional antibiotics. This study evaluated the therapeutic potential of a temperate, uncharacterized phage preparation induced from the *S. aureus* reference strain ATCC 25,923 using mitomycin C (MitC) for topical treatment of superficial MRSA-infected wounds in a rabbit model, both alone and combined with vancomycin. Key wound healing markers (collagen 1, MMP1, PDGF, FGF2) and inflammatory cytokines (IL-1, IL-6, TNF-α) were assessed alongside immunohistochemical analysis of IL-6 and TNF-α expression. The induced lysogenic phage demonstrated lytic activity against MRSA, resulting in a wound healing rate of 87.89%, which increased to 93.63% with combined vancomycin treatment. Enhanced healing correlated with elevated collagen, PDGF, and FGF2 expression and significant downregulation of pro-inflammatory cytokines (*p* < 0.0001). Immunohistochemistry confirmed decreased IL-6 and TNF-α expression in wound inflammatory cells. These findings provide preliminary in vivo evidence supporting phage-antibiotic synergy for treating MRSA wounds. Although promising, the phage preparation remains uncharacterized, limiting reproducibility and translational readiness. Further research is needed to characterize active phage components, evaluate safety, and optimize therapeutic protocols before clinical application. This study pioneers the in vivo use of temperate phages combined with antibiotics for MRSA wound treatment, addressing antibiotic resistance issues by improving wound closure, promoting healing factor expression, and reducing inflammation. These results lay an important foundation for developing novel therapies against resistant bacterial infections.

**Supplementary Information:**

The online version contains supplementary material available at 10.1038/s41598-025-34474-6.

## Introduction

The development of new antibiotics has diminished over the last few years, with a few companies remaining active in these domains^1^ The growing issue of antimicrobial resistance has reinvigorated interest in bacteriophage therapy as a potential alternative to antibiotics. These bacterial viruses may soon become one of the only remaining alternatives for treating bacterial infections^2^.

Virulent (lytic) phages are unable to integrate their DNA into the genome of the bacterial host. In contrast, temperate (lysogenic) phages can exist as extrachromosomal DNA or be incorporated into the bacterial host’s genome. When the phage DNA is integrated, it is referred to as 'prophage,' and the bacteria hosting it are termed ‘lysogenic’^3^. Temperate phages remain inactive within the cell unless they encounter a stressor. The reawakening of quiescent phages is known as induction, and it can occur spontaneously or as a result of several DNA-damaging processes, such as UV radiation or antibiotics. These prophages can transition into the lytic cycle, leading to the production of infectious phage particles that contain all the necessary functional and structural genes for genome excision, replication, and assembly of phage particles. This causes the bacterial cell to lyse (break apart), releasing additional phages^4^.

Phages are usually administered in conjunction with antibiotics since they must demonstrate efficacy alongside the standard of care, which is antibiotics. These approaches led to the discovery of phage-antibiotic synergy (PAS)^5^. Temperate PAS refers to a temperate bacteriophage’s potential to improve antibiotic efficacy against bacterial infections. Bacteria can grow more vulnerable to antibiotics after being infected with a temperate phage, according to studies^6–15^. Phages can increase bacterial susceptibility to antibiotics through several mechanisms that enhance antibiotic penetration and effectiveness. Phage adsorption and infection cause disruption or remodeling of the bacterial cell surface and membrane structures. This can increase membrane permeability, allowing antibiotics better access to intracellular targets that might otherwise be shielded. Moreover, Phage selective pressure selects for receptor mutants that escape infection but often restores antibiotic sensitivity by disrupting resistance mechanisms (e.g., efflux pumps or cell wall modifications)^16,17^. These mutations may simultaneously impair bacterial resistance pathways, re-sensitizing bacteria to antibiotics^18^.

Interestingly, some antibiotics, especially those targeting the cell wall (e.g., beta-lactams), may have synergistic effects when combined with phages, as phage-induced damage can enhance bacterial cell wall vulnerability, facilitating antibiotic action. Bacteriophages can disrupt biofilms, complex bacterial communities that are typically more resistant to antibiotics, thereby improving antibiotic penetration and bacterial clearance. Together, these combined actions make bacteria more permeable to antibiotics and restore or enhance antibiotic capacity to kill bacteria, particularly in resistant strains^8,19^.

Thus, PAS may help lower the amount of antibiotics required to treat bacterial infections by potentially minimizing the development of antibiotic resistance. This phage-antibiotic synergy is explored as a promising therapeutic approach against multidrug-resistant infections^19,20^.

Due to the significant functional and aesthetic role of skin tissue, treating skin wounds is a crucial study area. When the skin is damaged, bacteria can swiftly enter the underlying tissues and cause potentially fatal infections. The primary microbial strains found in patients with infected wounds include *Staphylococcus aureus* (*S. aureus*)*,* methicillin-resistant *Staphylococcus aureus* (MRSA), and *Pseudomonas aeruginosa.*^21–23^. MRSA can cause a variety of insults to patients in the surgical intensive care unit (SICU), including wounds through the skin and subcutaneous tissue, as well as invasive infections such as pneumonia, lung abscess, infective endocarditis, and sepsis as a community-acquired MRSA (CA-MRSA) infection, resulting in a longer hospital stay, morbidity, and mortality when compared to other organisms associated with hospital-acquired infections. It is a virulent organism that can vigorously invade skin and soft tissues causing cellulitis, necrotizing fasciitis, and diabetic foot ulcers^24^. Bacteremia caused by MRSA is frequently associated with 15–60% mortality rates and is characterized by strains that can partially tolerate vancomycin^25,26^. As a result, effective therapies are required to handle such pathological disorders^27^. Effective targeted treatments are still necessary because of the unique biological, non-sterile wound environment and the extremely intricate wound healing system^28^.

PAS is relevant and beneficial not only in systemic infection control but also specifically in local wound management, where it enhances healing by combining direct antibacterial effects with improved tissue repair and reduced inflammation. Clinical and experimental evidence in MRSA-infected wound models showed that phage-antibiotic combination promotes wound healing by reducing bacterial colonization and modulating inflammatory responses, thus accelerating wound closure and improving healing biomarkers^29,30^.

In a previous study by our research group, we isolated and characterized lytic phages with a high broad host range specific to MRSA from domestic sewage water at our tertiary care hospital at Theodor Bilharz Research Institute, Giza, Egypt^3^. Moreover, prophages were induced from a MitC-treated culture of *S. aureus* strain ATCC 25,923. As a continuation of our research in this field, this work aimed to evaluate the possible synergistic role of phage–antibiotic combinations, in an in vivo study through topical application onto a full-thickness skin wound infected with MRSA in a rabbit model.

## Results

### Bacterial isolates and vancomycin sensitivity test

**Fig. 1:**
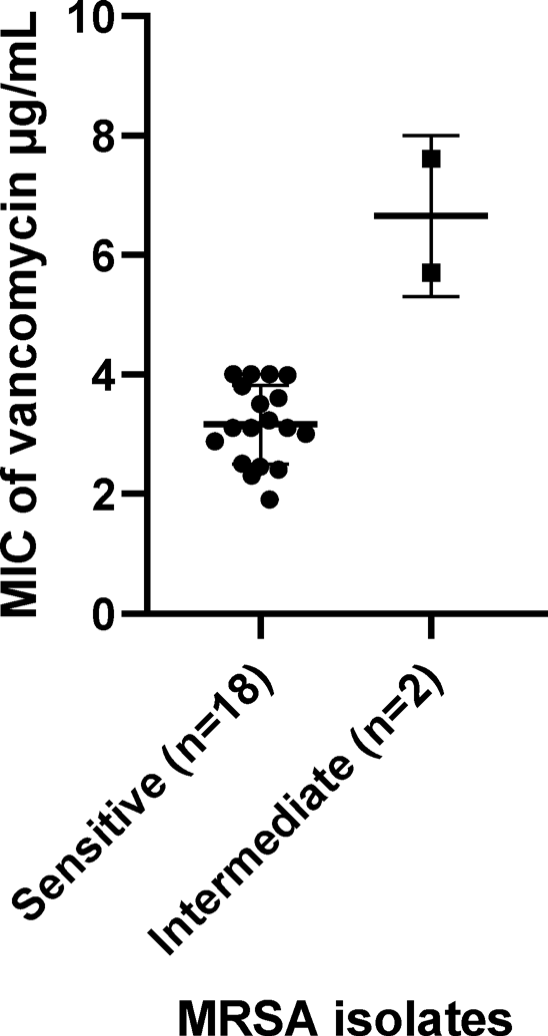
The Minimum Inhibitory Concentration (MIC) of vancomycin against MRSA of sensitive (n = 18) and intermediate (n = 2) isolates.

No Methicillin-resistant strains of *S. aureus* (n = 20) were found to be resistant to vancomycin as measured by NCCLS standard disk diffusion interpretive criteria for vancomycin (organisms for which the MIC of vancomycin is ≤ 4 µg/ml are considered susceptible) Fig S1. The mean values of MIC of vancomycin in tested MRSA isolates were statistically comparable (*p* = 0.11). The MIC of vancomycin against MRSA is presented in Fig. 1.

### Phage host range and efficiency of plating (EOP) analysis

**Fig. 2:**
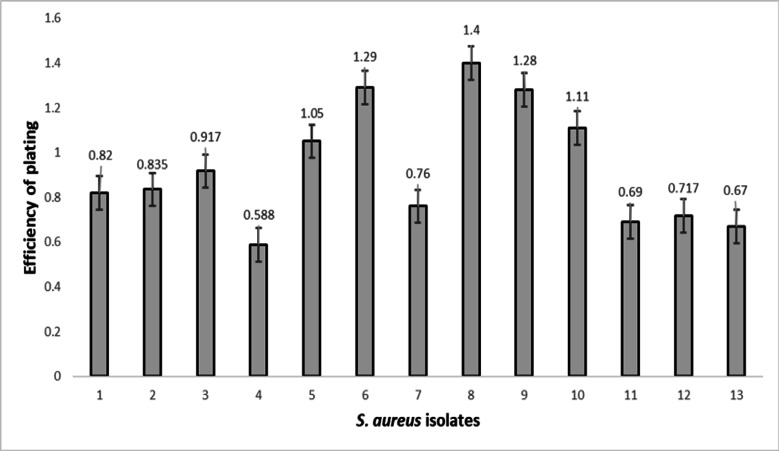
The efficiency of plating (EOP) relative to the number of plaques produced in *S. aureus* strain ATCC 25,923. The EOP was evaluated for 12 isolates of MRSA. The numbers on the vertical lines (y-axis) indicate EOP values, and the numbers on the horizontal line (x-axis) indicate the *S. aureus* isolates. Sample 1 is the reference sample (*S. aureus* strain ATCC 25,923). The results presented are the average of three technical assays. The EOP values were presented as the mean of three independent measurements followed by their standard deviation.

We evaluated the host range using a spot test with undiluted phage stock with a titer of 1 × 10^10^ PFU. Host range analysis showed that 12 out of 20 (60%) of the investigated MRSA were susceptible to phages, and the EOP for these 12 isolates was 100%—that is, under optimum conditions, any phage particle that attaches to a host cell can penetrate and form a plaque on the relevant isolates Table S1. The EOP values were presented as the mean of three independent measurements, along with their standard deviation. Investigation indicated that EOP values in the tested MRSA isolates varied from 0.588 to 1.28 Fig. 2.

EOP = (plaques on test strain)/(plaques on reference ATCC 25,923) at fixed MOI. Reference strain EOP is defined as 1.0 (100%); values ≥ 1.0 indicate equivalent or superior plating efficiency, not literal 100% particle success due to adsorption/ burst variations." ATCC 25,923 is plotted as Sample 1 at EOP = 1.0 ± SD.

### Checkerboard analysis

We studied the cumulative effect of the induced phages with vancomycin. Combining phages and vancomycin reduced the MIC by multiple folds compared to the individual effects. For vancomycin, the MIC in the individual treatment was 5 μg/mL, while the MIC in combination with a fixed concentration of phage (10^8^ PFU/mL) was 1.25 μg/mL. FIC for vancomycin was 0.25. Additionally, the MIC of the phage alone was 10^8^ PFU/mL, and in combination with a fixed concentration of vancomycin (10 µg/mL) was 10^5^ PFU/mL. FIC for the phage was 0.001. The FICI of the vancomycin in combination with phage was 0.251. Thus, the results of the checkerboard analysis showed that the phage and vancomycin in combination had a synergistic effect (FICI ≤ 0.5)**.**

### In vivo wound healing and wound contraction percentage

**Fig. 3:**
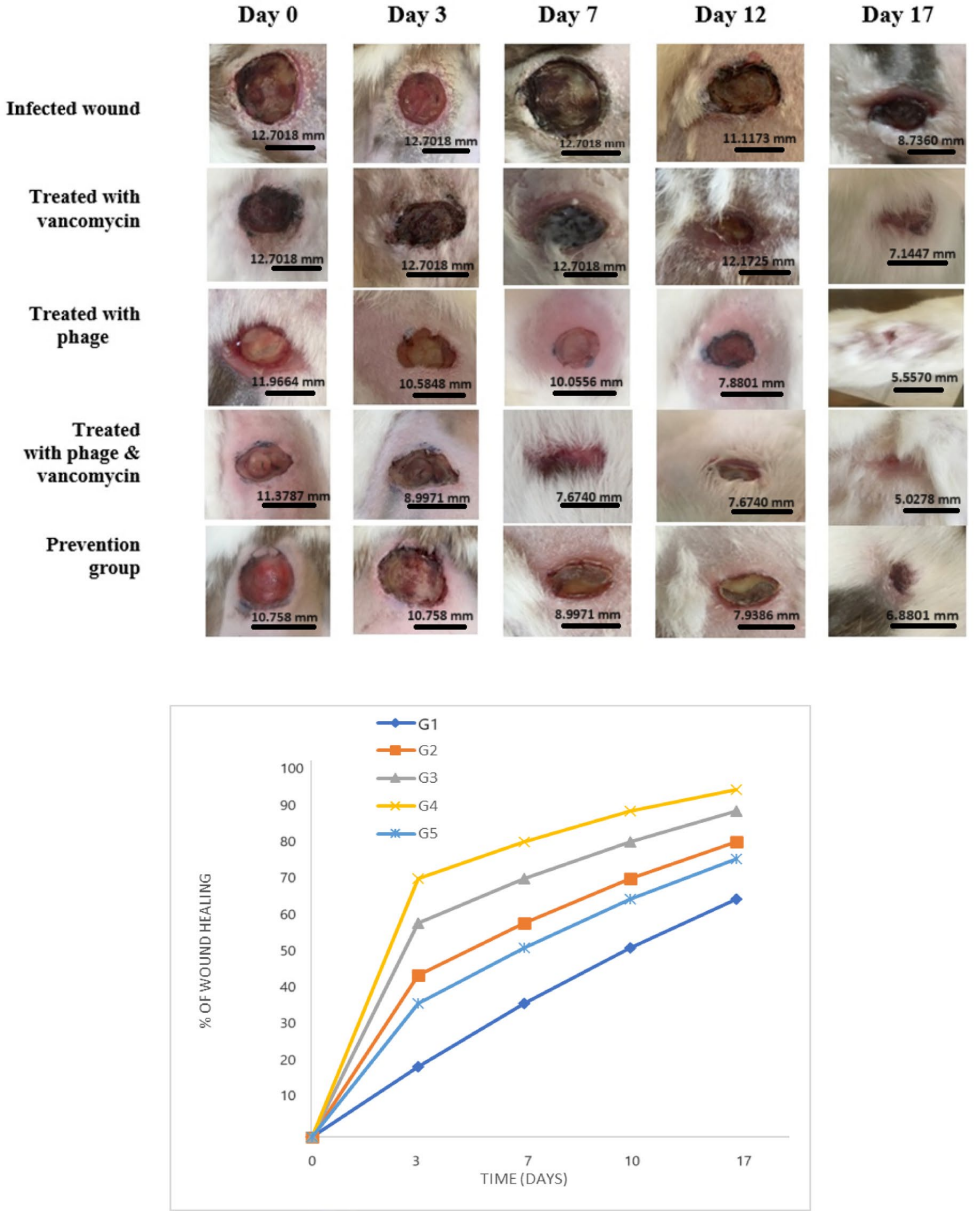
Analysis of full-thickness incisional infected wounds in rabbits by millimeter (mm) at a fixed focal distance. The non-treated infected wound (negative control; G1) (n = 3), infected wound treated with vancomycin (G2) (n = 3), infected wound treated with phage (G3) (n = 3), infected wound treated with phage & vancomycin (G4) (n = 3), and the prevention group (G5) (n = 3), from day zero till day 17 (*p* < 0.0001).

The wound closure percentage throughout 17 days indicates that the combination of phages and vancomycin is promising against MRSA and is superior to that of phage-only and vancomycin-only treatments, with good wound contraction percentage in a full-thickness wound infection in a rabbit model Tables S2 & S3. The results shown in Fig. 3 display the percentage of wound closure, which is critical for successfully repairing wound tissues. By day 17, the phage-vancomycin group showed a higher percentage of wound closure (93.63%). On the other hand, the infected wound group that applied sterile gauze showed a lower percentage of wound closure (64.01%), resulting in non-healing wounds with a purulent appearance. In addition, the vancomycin-treated group showed (79.61%) of wound contraction. The prevention group exhibited a lower percentage of wound contraction (74.84%), compared to the group treated with phage (87.89%). Multiple comparisons conducted via One-Way ANOVA between the infected group and various treated groups indicated statistical significance in G2 (*p* = 0.0134), G3 (*p* = 0.0003), and G4 (*p* < 0.0001). However, no significant difference was observed in comparison with G5.

### Histopathological analysis of wound healing and IHC

**Fig. 4:**
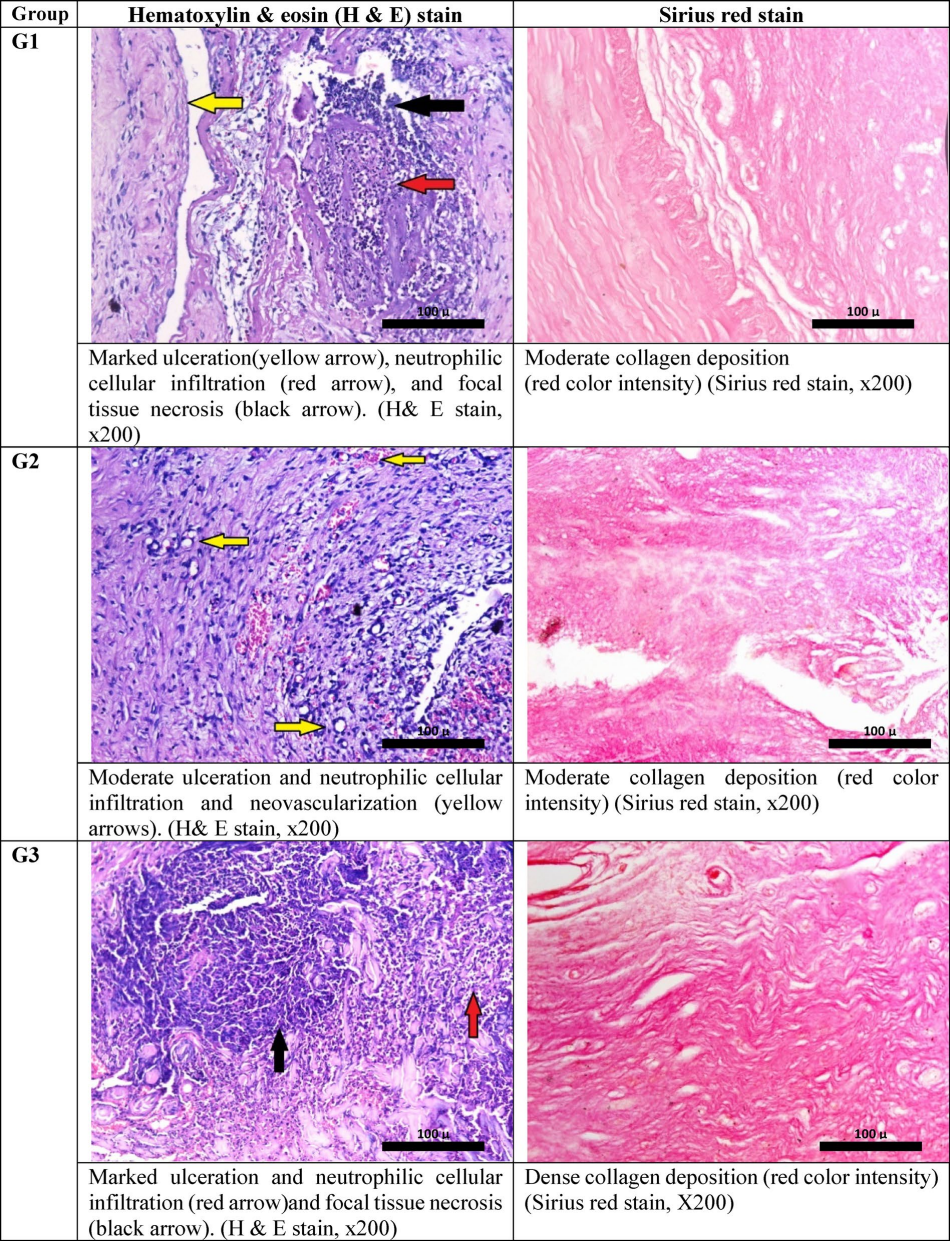

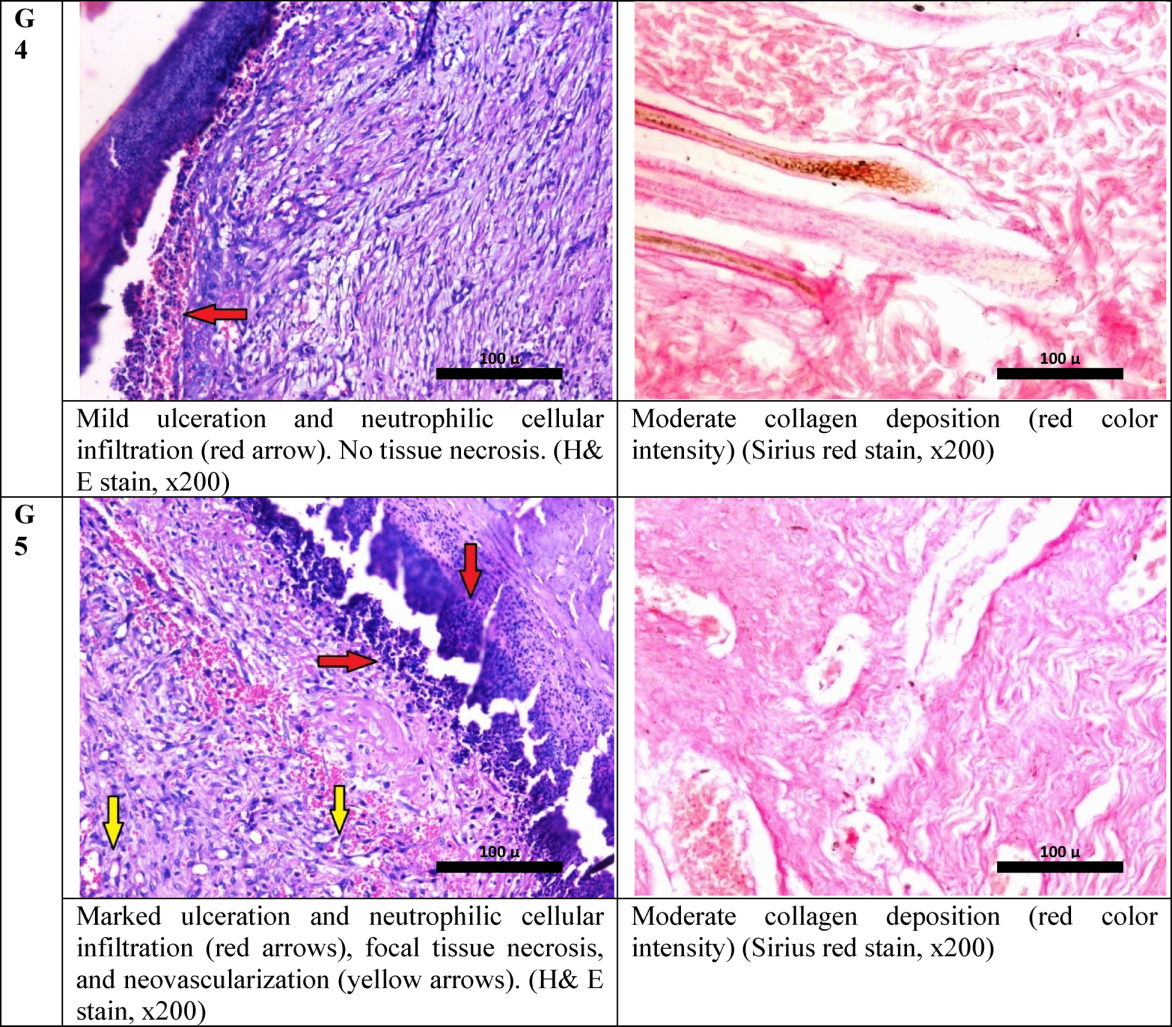
Histological aspects of wounded skin and evaluation of collagen deposition in studied groups after 17 days.

Histopathological examination of sections prepared from the wound edges of different studied groups revealed marked ulceration and polymorphonuclear cellular infiltration in the skin lesion of the infected non-treated group of rabbits with focal tissue necrosis and moderate fibrosis Fig. 4.

In Table 1. the wound of rabbits treated with vancomycin alone showed less ulceration and inflammation compared to the control infected rabbit, while the wound of the rabbit treated with vancomycin and the phage showed the best healing response, with the least ulceration and inflammation compared to all other studied groups. On the other hand, both phage phage-only treated group and the prevention group showed unremarkable changes compared to the control infected group.

**Table 1:** The FIC/FICI results from the checkerboard assay.

Component	MIC alone	MIC in combination	FIC
Vancomycin	5 μg/mL	1.25 μg/mL	0.25
Phage	10^8^ PFU/mL	10^5^ PFU/mL	0.001
FICI	–	–	0.251

MIC, Minimum inhibitory concentration; FIC, fractional inhibitory concentration; FICI, fractional inhibitory concentration index.

**Fig. 5:**
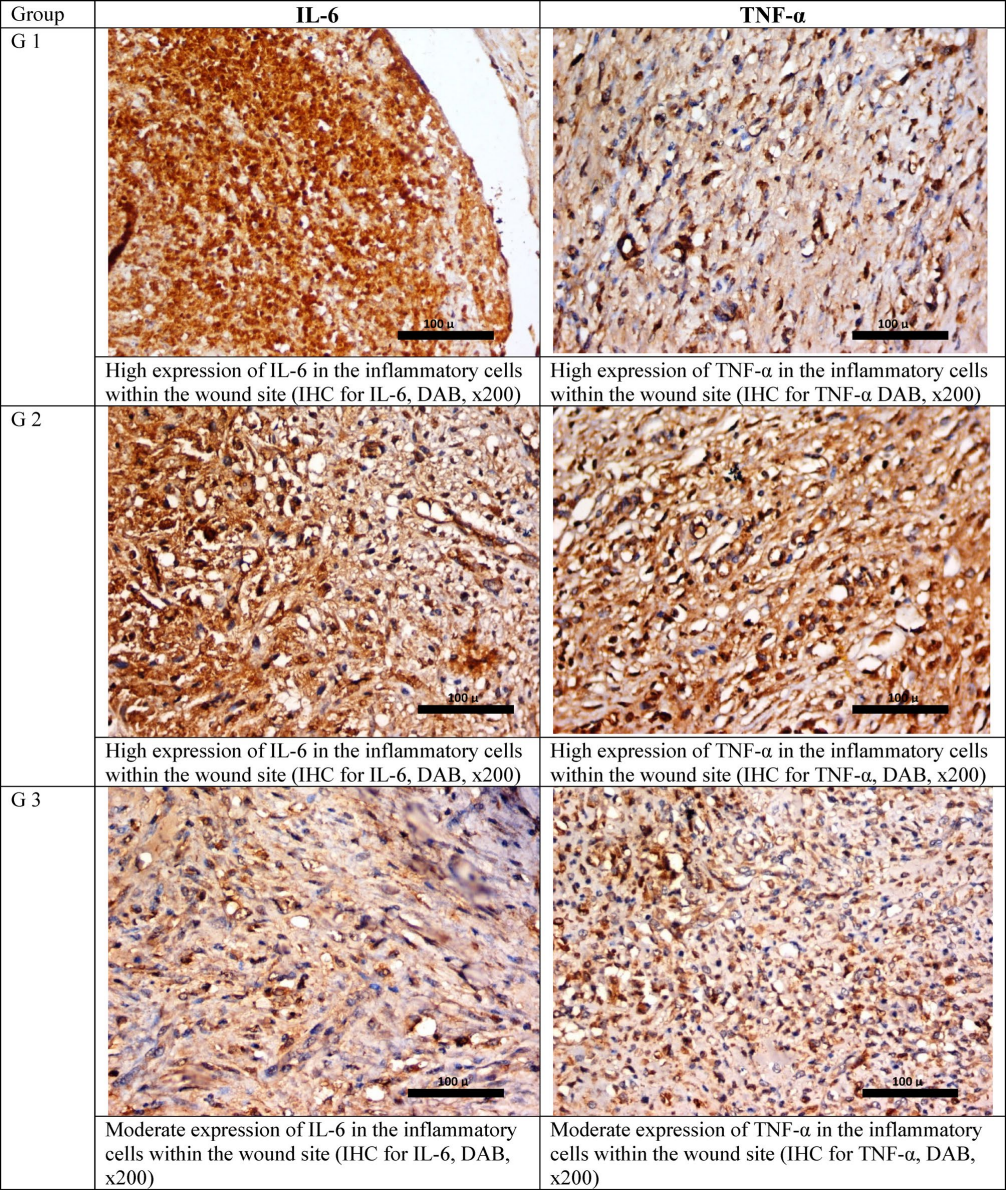

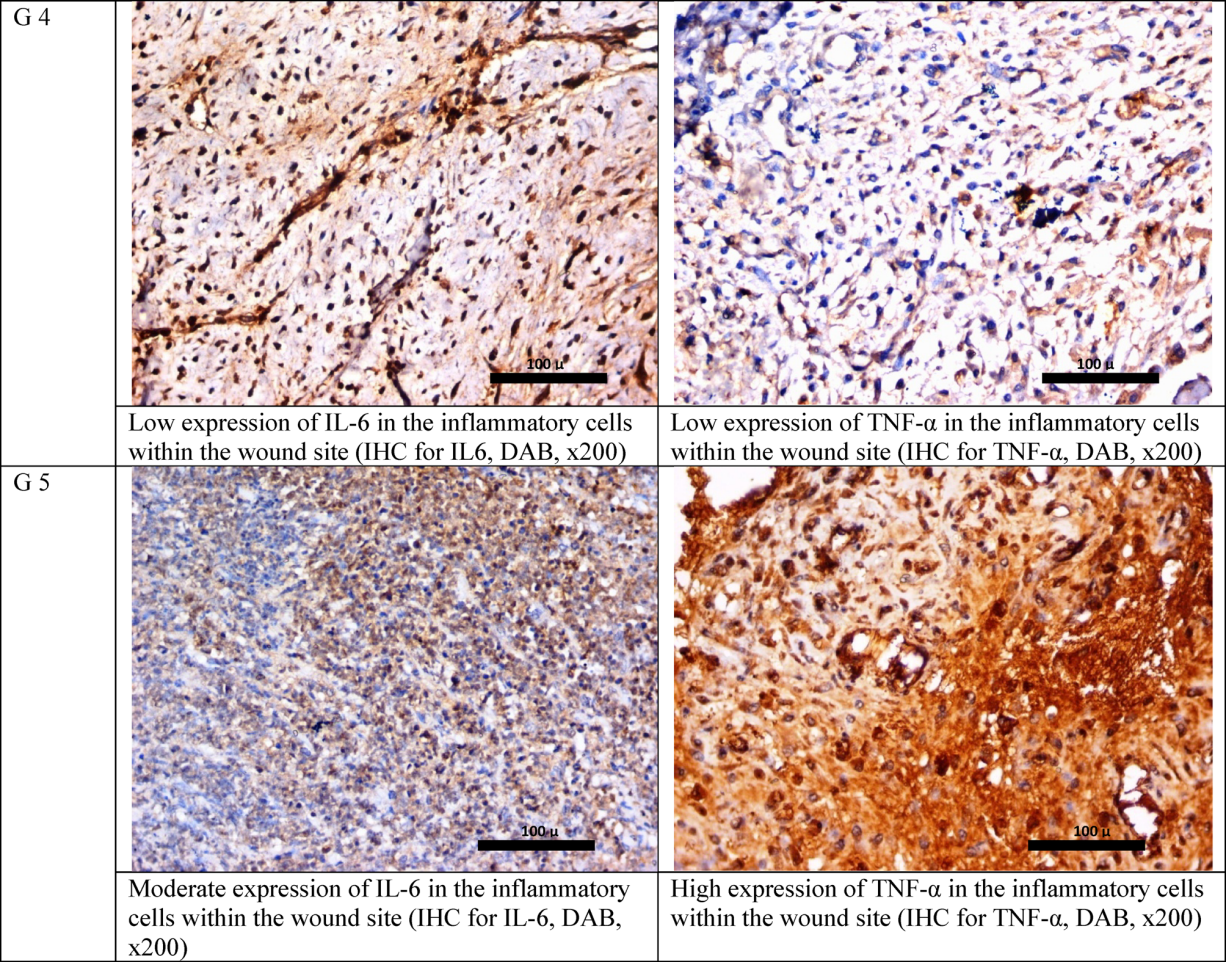
IHC staining for IL-6 and TNF-α in the studied groups after 17 days.

As regards the immunohistochemical study, it was found that IL-6 and TNF-α showed the lowest expression parameter in the vancomycin and phage-treated rabbit, while the control infected group and the vancomycin-treated group showed higher expression of IL-6 and TNF-α. The phage-only treated groups showed moderate expression of both IL-6 and TNF-α Fig. 5.

### Molecular analysis of wound healing

**Fig. 6:**
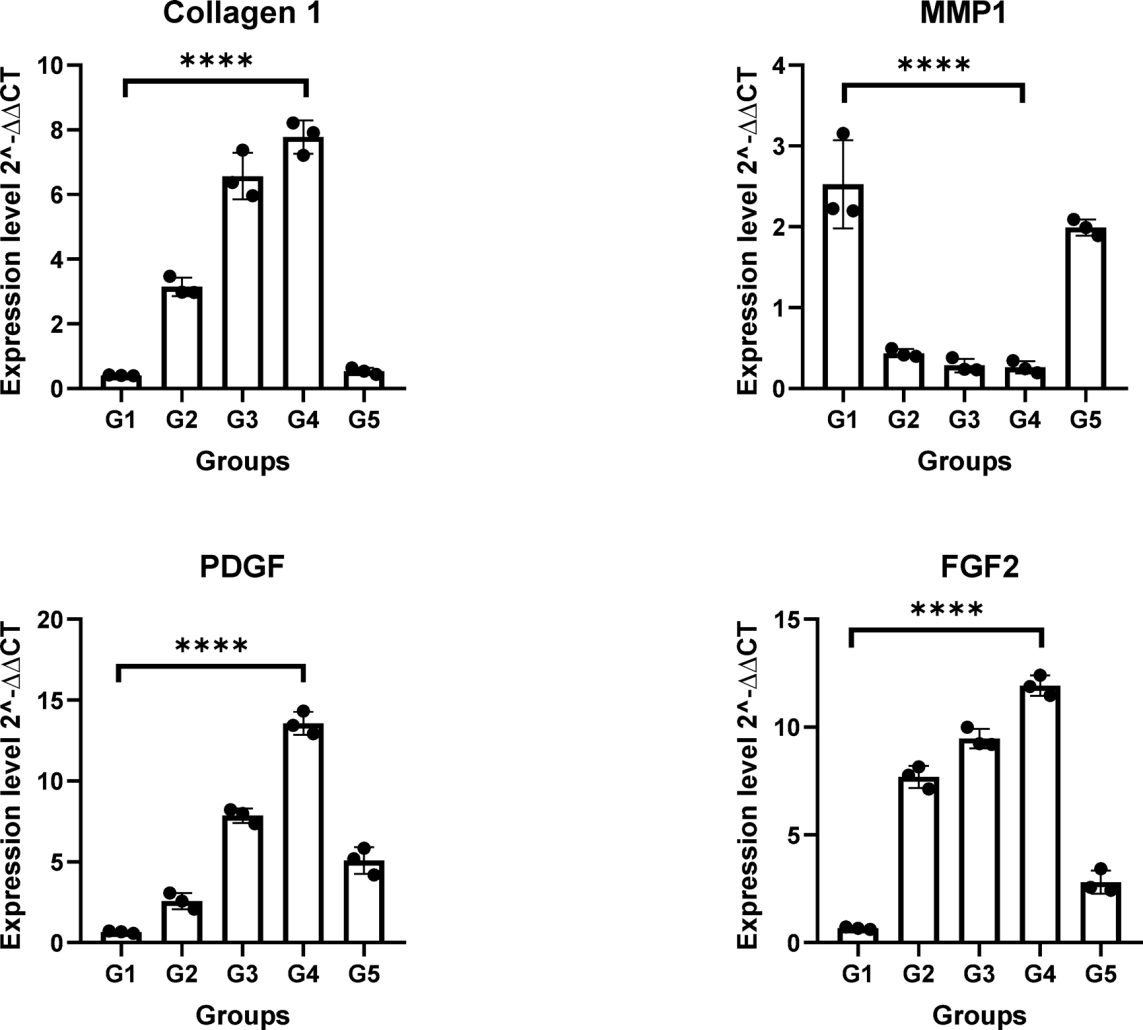
Collagen 1, MMP1, PDGF, and FGF2 mRNA levels among the studied groups. All experiments for each gene were conducted in technical triplicates. Rabbit groups were conducted as independent biological triplicates. ****p < 0.0001 is considered significant.

**Fig. 7:**
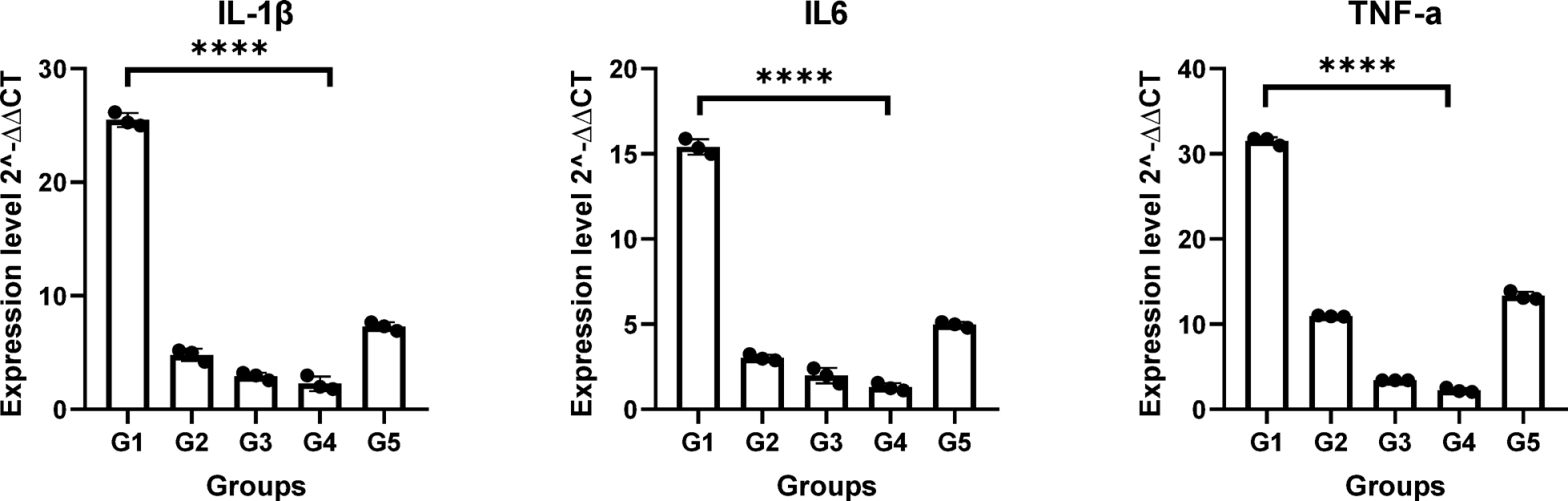
IL-1β, IL-6, and TNF-α mRNA levels among the studied groups. All experiments for each gene were conducted in technical triplicates. Rabbit groups were conducted as independent biological triplicates. ****p < 0.0001 is considered significant.

Using qPCR, we assessed the pro-inflammatory cytokines IL-1, IL-6, and TNF-α in blood samples, which play important roles in tissue repair and wound healing processes. Additionally, collagen type 1, Matrix Metalloproteinase 1 (MMP1), Platelet-Derived Growth Factor (PDGF), and Fibroblast Growth Factor-2 (FGF-2) were also assessed in wound tissue, all play important roles in the wound healing process, including infected wounds. Figure 6. shows that as the wound progresses towards healing, the expression of collagen 1 increases and the expression of MMP1 typically decreases promoting proper wound healing and collagen deposition. PDGF and FGF-2 are significantly increased as wound healing progresses. Thus, supporting the tissue remodeling and restoration of normal tissue architecture (*p* < 0.0001). Figure 7. shows that in G4 downregulation of IL-1, IL-6, and TNF-α was observed compared to G1 in which these genes were upregulated in infected untreated wounds, to enhance the immune response and aid in the clearance of the bacteria. Their decreased levels in wounds progressing toward healing are important for the resolution of inflammation and the progression of healing (*p* < 0.0001).

## Discussion

Bacteria evolve resistance through antibiotic overuse/misuse via selective pressure favoring resistant mutants (e.g., target modification, efflux)^16–18^. Numerous research investigations have revealed that phages have extraordinary success in eradicating MRSA biofilms and free-floating cells due to their abundant occurrence in nature, their selectivity towards target bacteria, and their safety^3,31–37^.

Mitomycin C induction is a common approach for inducing prophages in bacterial cultures. It successfully initiates the lytic cycle of temperate phages, leading to the liberation of phage particles and the destruction of the host bacterial cells^38^.

The *S. aureus* ATCC 25,923 strain harbors dormant prophages integrated into its genome. *S. aureus* strain ATCC 25,923 (NCBI Reference Sequence: NZ_CP009361.1) contains two distinct prophage elements related to Peeveelviruses tp3101 (1,513,010–1,581,201) and PT1028 (345,743–379,383). These prophages are intact and may be capable of induction as functional temperate phages^39^. Generally, the host range of temperate *Staphylococcus* phages tends to be narrow^40^. The observed host range in this study indicates that both phages might be present or that one is mostly responsible for the activity. Here, despite its importance, we didn’t characterize individual phage contributions using techniques such as PCR and sequencing which can differentiate between the two phage genomes^41,42^. Our primary focus in this study was to investigate temperate phages as potential alternatives. Additionally, We aimed to explore the synergy between temperate phages and antibiotics. This aspect of our research is critical, as combining phage therapy with existing antibiotics could enhance treatment efficacy against resistant bacterial strains. The potential for these phages to work synergistically with antibiotics presents a promising avenue for combating bacterial infections^43,44^.

When *S. aureus* ATCC 25,923 is treated with mitomycin C, a DNA-damaging agent, these prophages are triggered to enter the lytic cycle, where they multiply within the bacterial host, ultimately causing cell lysis and releasing new phage particles. As a result, ATCC 25,923 undergoes self-lysis due to the activation of its own prophages. Bacteria may exhibit homoimmunity to their own prophages, meaning they are resistant to reinfection by the phages they produce due to the presence of repressor proteins that block the initiation of the lytic cycle. However, in this study, *S. aureus* ATCC 25,923 did not display homoimmunity against its induced phages, and once released, the phages reinfected and successfully propagated within the same bacterial strain. Plaque assay confirmed the phages’ ability to infect and lyse ATCC 25,923 cells, and the presence of plaques demonstrated effective bacterial cell destruction. Despite carrying prophages, ATCC 25,923 remains susceptible to them following induction with mitomycin C, allowing the phages to reinfect and eliminate the bacterial cells. This discovery underscores the potential of induced prophages as a promising approach for combating bacterial infections^3,45^.

We did not detect any vancomycin-resistant *Staphylococcus aureus* (VRSA) isolates, i.e., all the studied bacteria were MRSA. The efficiency of phage replication was detected by the EOP method which indicated that EOP values in the tested MRSA isolates varied from 0.588 to 1.28. EOP values closer to 1 would be desirable^46^. Checkerboard results showed that the capacity of phages and antibiotics in combination to eliminate the bacterial burden is superior to that of phage-only and antibiotic-only treatments. This result is similar to the results obtained by previous research^44,47^.

One of the most applied wound healing models is the incisional wound, which is supposed to resemble acute clinical wounds. The wound area can be observed over time by taking regular images, and the wound closure rate is calculated by comparing the size of the wound to its initial measurements^27,48^. Interestingly, the findings in our study demonstrated that the combination of phages and antibiotics (PAS approach) against infected wounds with MRSA, had a greater ability to heal the wound than either treatment alone (phage or antibiotic). This is in concordance with many studies that have shown that phage-antibiotic combinations are more efficient than phage-alone treatments^43,49^. Luo et al*.*^50^ found that Phage pB3074 combined with cell wall-targeting antibiotics could produce a synergistic antibacterial effect in vitro bactericidal activity. Mengshi Zhao et al*.* (2024) demonstrated phage-antibiotic synergy (PAS) and showed considerable broad-spectrum antibacterial potential against different clades of clinical MDR *Klebsiella pneumoniae* pathogens. The improved treatment outcomes of optimized PAS were also evident in a murine model, where mice receiving optimized PAS therapy demonstrated a reduced bacterial burden in mouse tissues^51^.

In our rabbit wound model, the combination of phages and vancomycin accelerated wound closure than either treatment alone. This aligns with findings from Bahareh Lashtoo Aghaee et al*.*^52^, which indicated that phages can lower the minimum inhibitory concentration (MIC) of antibiotics for resistant strains. 

In the rabbit model, in the initial stages of wound healing, IL-1, IL-6, and TNF-α inflammatory cytokines were upregulated to initiate the healing process. These cytokines are secreted by different cells, such as immune cells, fibroblasts, and endothelial cells, in response to tissue injury. They play key roles in intervening in inflammation, immune response, and tissue repair. As the wound proceeded towards healing and the infection started to be resolved, the expression of these cytokines decreased. They play a crucial role in the initial inflammatory period of wound healing by stimulating the recruitment of immune cells and initiating tissue repair processes. As the wound resolved and tissue regeneration took place, the levels of them decreased, thus allowing the transition to the subsequent phases of healing and remodeling^53^.

The expression levels of PDGF, FGF2, and collagen 1 play an important role in wound healing because they promote cell proliferation, the development of new blood vessels, and the generation of extracellular matrix components. PDGF and FGF2 boost the movement and proliferation of cells such as fibroblasts, which are responsible for producing collagen. Furthermore, PDGF promotes angiogenesis, the formation of new blood vessels that supply nutrition and oxygen to the healing tissue. Increased PDGF and FGF2 expression in healed wounds as observed in G4 aids in tissue restructuring and the restoration of normal tissue structure, facilitating effective wound healing^54^. Collagen 1, the most prevalent form of collagen in the extracellular matrix of healthy skin, exerts an important role in wound healing. The expression of collagen 1 increased during this process in G2, G3, and G4. Collagen 1 serves as a major component of granulation tissue, which consists of new blood vessels, fibroblasts, and components of the extracellular matrix. It proposes structural support and contributes to restoring the integrity and strength of the healing wound, eventually leading to scar formation through tissue remodeling^55^.

The reduction in inflammatory cytokines and the marked increases in collagen 1 and FGF2 expression observed in the combination therapy group directly correlate with accelerated wound closure and more effective tissue regeneration. Compared to antibiotic monotherapy, the phage-antibiotic regimen not only resolves infection but also enhances the physiological healing cascade, yielding rapid restoration of skin architecture and function. These findings underscore the translational relevance of phage-antibiotic synergy, supporting its clinical value for advanced wound care^19,56^.

Several studies demonstrated that PAS enhances bacterial eradication, reduces inflammatory markers, and stimulates regenerative markers, leading to superior tissue repair compared to antibiotics alone. Single-agent topical vancomycin is increasingly considered outdated, as it often fails to sufficiently resolve inflammation or promote tissue regeneration, limiting therapeutic improvement and supporting antibiotic resistance. Combination therapy also minimizes the emergence of drug-resistant mutants, promotes wound contraction, and is less disruptive to host microbiota than antibiotic monotherapy^19,29,30,57^.

Moreover, studying the lytic activity of the induced prophages on the collected MRSA isolates in this study showed that only twelve out of twenty isolates were lysed. Thus, to improve the results of our study, we aim to start ongoing research for the enhancement of phage discovery and selection that may lead to the identification of highly effective phages against MRSA. This may involve the screening of various phage libraries or the development of engineered phages or phage lysis proteins with expanded bactericidal activity^20^.

Finally, our research adds to the increasing evidence that PAS is effective against MRSA in an animal model, emphasizing its promise as a flexible treatment option designed for particular pathogens and resistance patterns. According to a 2024 article in Frontiers in Pharmacology, employing simple animal models is essential for assessing the safety and effectiveness of PAS prior to clinical application^58^. Our rabbit model offers valuable insights into the use of PAS for treating wounds infected with MRSA.

The use of temperate phages raises concerns regarding lysogeny and potential horizontal gene transfer, which were not addressed in this study. In the case of *S. aureus*, pathogenicity islands like SaPIs are known to be mobilized by phages. Comprehensive genomic analyses of the phage preparations are required. Moreover, despite their risks, temperate phages offer some potential advantages in medical treatments. They are abundant and diverse, presenting a varied range of therapeutic agents that can be engineered for special applications. By altering them genetically, they can be converted into lytic phages, improving their therapeutic potential. Furthermore, temperate phages can disrupt biofilms and synergize with antibiotics, improving treatment outcomes. Advances in genetic engineering are necessary for utilizing these benefits while justifying the risks associated with temperate phages^59,60^. Future studies should focus on larger sample sizes and exploring different phage-antibiotic combinations to validate the therapeutic potential of temperate phage therapy against MRSA.

### Limitations, conclusion, and future perspectives

This study is limited by the use of an uncharacterized mixture of temperate phages induced from *S. aureus* ATCC 25,923, rather than a purified or defined phage preparation. This introduces variability due to the random nature of prophage induction, making exact replication and standardization challenging, as the precise identity and concentration of active phage components remain unknown. Additionally, the observed plaques in assays may result from the mobilization of multiple prophage elements, not solely the original induced phage, which complicates mechanistic interpretation without further characterization, such as sequencing of plaque isolates. The demonstration of phage-antibiotic synergy (PAS) was limited to a specific model and strain, so broader testing across diverse strains is needed to confirm generalizability. As the phage preparation is undefined, these findings should be considered proof-of-concept rather than a step toward clinical or regulatory development of a pharmaceutical product.

This study did not include a quantitative assessment of bacterial load in the wounds nor attempts to re-isolate active phages from the wound bed. Serial swabbing/ plating to confirm in vivo phage activity and persistence, and bacterial clearance dynamics, and they are planned as additional endpoints in future work essential for mechanistic validation. Also, no albumin pre-testing was performed. Phage stability in serum/wound exudate warrants future assessment via adsorption assays.

Moreover, our study lacks specific phage characterization (e.g., sequencing and phylogenetic placement), due to a focus on proof-of-concept PAS. This restricts insight into the phage diversity and mechanisms. But hopefully we plan to address this in future work.

Despite these limitations, our findings highlight the potential of induced lysogenic phage-antibiotic combination therapy as a promising approach for treating MRSA infections, with combination therapy showing enhanced wound healing and reduced inflammation compared to phage-alone treatment. Antibiotics can sensitize bacteria to phage attacks by inducing physiological stress responses that promote phage infection or reduce bacterial defenses. and the introduction of phage cocktails targeting multiple MRSA aspects, which are the diverse bacterial targets or virulence factors affected by phage therapy, may further improve efficacy and mitigate the risk of bacterial resistance. Advances in genetic engineering could also enable the development of phages with enhanced antimicrobial properties. Interestingly, the main challenge in clinical settings remains the effective management of MRSA-related sepsis, particularly in surgical wounds, which refers to systemic infection that originates from wound sites, a major clinical challenge these combined therapies aim to address. Early intervention using combined vancomycin and phage therapy may offer a valuable strategy, but further research—including detailed phage characterization, expanded in vivo studies, and clinical trials—is essential to validate the safety and effectiveness of this approach before widespread implementation.

Regarding the animal experiments, while the rabbit sample size is comparable to other published wound healing studies, future studies should consider larger cohorts to validate findings and confirm therapeutic efficacy across broader populations.

## Materials and methods

### Phages and host bacteria used in the work

#### Host bacteria MRSA isolates

Twenty methicillin-resistant strains of *S. aureus* (MRSA) were chosen for the study that were formerly collected in our previous research work published in 2022 and stored in the form of glycerol stocks at − 70 °C^3^.

#### Vancomycin sensitivity test& MIC determination for MRSA to vancomycin

Minimum Inhibitory Concentrations (MIC) of the MRSA isolates to vancomycin (10 mg/L stock solution dissolved in nuclease-free water) were determined according to Zaki WK and Hager R^61^. The growth inhibition zones were carefully measured with calipers and recorded according to the standard Kirby-Bauer disc diffusion method and the National Committee for Clinical Laboratory Standards (NCCLS) guidelines. MIC determination was performed in triplicate. *Staphylococcus* spp. for which the MIC of vancomycin is ≤ 4 µg/mL is considered susceptible. Isolates for which the MIC is 4–8 µg/mL are intermediate, and those for which the MIC is ≥ 16 µg/mL are resistant^62^. During the test, the control strain *S. aureus* ATCC 29,213, which is well-characterized and has a known susceptibility profile to vancomycin, was used to ensure the accuracy and reliability of the test results.

### Induction and preparation of bacteriophage suspension

Bacteriophages were induced using our previously published procedure^3^. Phage suspension was prepared by centrifuging the induced culture of *S. aureus* reference strain ATCC 25,923, at 4000 rpm for 20 min at 4 °C to remove unlysed bacterial cells. The supernatant was gently removed and filtered using a 0.2 μm pore-size syringe filter to remove bacterial debris. After that, the filtered phage suspension was used immediately for host range determination, and the rest was stored at 4 °C for further characterization and analysis.

### Spot test and host range determination

To determine the efficacy of the filtrate acquired from the induced culture of *S. aureus* reference strain ATCC 25,923 with MitC against MRSA isolates. According to our previously published protocol and under aseptic conditions, MRSA isolates were cultured in LB broth overnight at 37 °C and then sub-cultured by the addition of 5 mL LB broth to 50 µl from overnight culture and grown at 37 °C for 3 h. One hundred µl of 3 h culture was inoculated into 3 mL of molten top agar overlaid onto LB agar plates. Each overlay was allowed to solidify for 30 min. Spots of 10 µl of the phage lysate were spotted onto the MRSA isolates overlay, dried, and then incubated at 37 °C overnight. Results were analyzed visually based on the detection of any lysis^3^.

The EOP was tested for MRSA isolates that were positive in the host-range spot-test assay (occurrence of a clear lysis zone). EOP values were calculated as the ratio of lysed plaques produced in each susceptible isolate for a fixed dose of phage (10 µl of dilution 10^5^) divided by the number of plaques produced in *S. aureus* strain ATCC 25,923^63^.

### Phage propagation and titration

To obtain an enriched phage lysate, according to our previous study protocol^3^, phage lysate was propagated through three successive enrichment cycles by inoculating 50 µl of it into a 10 mL LB broth of a grown cocktail of MRSA bacterial culture (OD_600_ = 0.4). The choice of these strains was based on their susceptibility to the phages, which was proved in spot test and host range determination. After one hour, the culture was centrifuged at 4000 rpm/20 min/4 °C, then passed through a 0.22 μm sterile syringe filter obtained from Thermo Fisher Scientific. Phage titration was carried out using the double agar overlay technique^3^.

Plaques were counted as areas of clearing in bacterial lawns following an overnight incubation. The multiplicity of infection (MOI) was determined using the formula:

$${\text{Phage titer }}\left( {{\mathrm{PFU}}/{\mathrm{mL}}} \right) \times {\text{phage volume}}/{\text{colony forming unit }}\left( {{\mathrm{CFU}}/{\mathrm{mL}}} \right) \times {\text{bacterial volume }}\left( {{\mathrm{mL}}} \right)$$

### In vitro study of vancomycin- phage combination: Checkerboard assay

The assay was used to assess the combined effect of vancomycin and phages on MRSA growth. A 100 µl series of two-fold dilutions of the vancomycin starting from 40 µg/mL to 0.156 µg/mL were prepared in a 96-well microtiter plate. Each well contained a different concentration of the antibiotic. Similarly, a 100 µL of phage dilutions starting from 10^2^ to 10^9^ were prepared in SM buffer (50 mM Tris–HCL pH7.5, 100 mM NaCl, 8 mM MgSO_4_, 0.01% gelatin v/w) in another microtiter plate. Combination wells were prepared using fixed concentrations of the antibiotic that were added to each well of the phage dilution plate and vice versa. All wells were inoculated with a consistent bacterial inoculum of 100 µl of cultures grown to an OD_600_ of 0.2 (logarithmic growth phase). Control wells contained only bacteria (no antibiotic or phage) as positive growth controls. Additionally, wells with only sterile growth media as negative controls were included.

Synergy testing was performed in triplicate. After 18 h of incubation, the optical density was measured using a microplate spectrophotometer, followed by calculating the percent of growth measurements as follows: (OD treatment − OD growth control/OD growth control) * 100.

The fractional inhibitory concentration index (FICI) for each combination was analyzed to determine the synergy between the vancomycin and phage. The FICI is the sum of the fractional inhibitory concentration (FIC) of the antibiotic and the FIC of the phage. The FIC of each component is determined by dividing the minimum inhibitory concentration (MIC) of the combination well by the MIC of the antibiotic or phage alone.

FICI ≤ 0.5 indicates synergy between the antibiotic and phage. FICI > 0.5 and ≤ 4 indicates no interaction (additive effect). FICI > 4 indicates antagonism (the antibiotic and phage interfere with each other’s activity)^64^.

### Pre-clinical trial: constructing the infection model and administering therapy

#### Selection of experimental animals and sample size

The preclinical study protocol (PT: 588) was approved by the TBRI Institutional Review Board under Federal Wide Assurance (FWA00010609), and the work was conducted in accordance with the World Medical Association’s Code of Ethics for Experiments with Animals.

Since rabbits are commonly used for studying wound healing, fifteen male New Zealand White rabbits (9–11 weeks old), weighing approximately 2–2.5 kg, were selected for the in vivo animal study. The animals were purchased from the animal house at TBRI. These strains are commonly used for research activities. They are less aggressive and have fewer health problems compared with other breeds^65^. They were divided into five groups, each containing three rabbits Table 2.

**Table 2:** Description of the groups of rabbits included in this study.

Studied group	Treatment regimen
G1	Challenged with MRSA bacteria only (no treatment, control group)
G2	Challenged with MRSA bacteria, then after 2 h vancomycin was applied
G3	Challenged with MRSA bacteria, then after 2 h phage was applied
G4	Challenged with MRSA bacteria, then after 2 h phage & vancomycin were applied
G5	Received phage, then after 2 h, were challenged with MRSA (prevention group)

G1: Group1, G2: Group2, G3: Group 3, G4: Group4, and G5: Group5.

#### Maintenance of rabbits in the animal house, diet, and handling

Daily regular contact with newly arrived rabbits in the animal house was done to reduce stress during handling. As a period of acclimatization to the surroundings and the daily routine in the animal quarters, rabbits were quarantined for two weeks. The vet also examined the most common diseases, such as scabies, during this time. Rabbits were kept individually in stainless-steel cages hung at a height of 0.8 cm from the ground so that excrement could fall into collecting trays. The cages used met institutional animal welfare standards. The rabbit cages were made of a non-toxic plastic floor for easy cleaning and to prevent pressure sores. The nesting material was wood shavings, and bedding was provided on top to ensure comfort and enrichment for the rabbit. They were allowed free access to a standard laboratory diet and mineral water ad libitum. It was used as the sole drinking source to avoid potential contaminants.

### In vivo phage treatment

#### Surgical procedure of full-thickness wound model (skin infection model)

The surgical procedures for the full-thickness incisional cutaneous wound were performed according to a previously published study^66^. Before any procedures, shaving of the back hair of the dorsal area was done using an electric shaver, and the shaved hair was cleaned away to avoid contamination. Rabbits were premedicated by 5 mg/kg intramuscular injection of Meperidine and received inhaled anesthetics in the form of 6% Desflurane in 100% oxygen individually in an anesthesia gas polypropylene box. Desflurane is one of the newest inhalation anesthetics that’s characterized by a rapid onset of both induction and recovery of anesthesia, which gives it superiority to other inhalation anesthetics^67–69^. Applying topical local anesthetics was not performed, as they alter the process of wound healing, which will have implications on the outcome of the study^70^. A full-thickness circular wound with a diameter of 2 cm was formed on the dorsal area of the rabbit using dissecting scissors and sterile forceps. The pain caused by the surgical incision was antagonized by paracetamol 200 mg/Kg added to drinking water^71^. Pain management followed institutional guidelines: rabbits were premedicated with 5 mg/kg intramuscular Meperidine and maintained under 6% Desflurane anesthesia during surgery. Post-incision pain was managed with 200 mg/kg paracetamol administered orally via drinking water^72^. Animals were monitored daily for welfare, including wound appearance, behavior, and body weight; humane endpoints included > 20% weight loss, non-weight-bearing lameness, or unresponsive infection after 10 days. The primary endpoint was wound closure percentage at day 17, assessed via daily photography and ImageJ analysis. Humane endpoints included > 20% body weight loss, severe lethargy, or non-healing purulent wounds after day 10, triggering early euthanasia; no animals reached these criteria. Rabbits were euthanized humanely per TBRI protocols, and tissues were harvested. All procedures complied with Animals in Research: Reporting In Vivo Experiments (ARRIVE) guidelines^73,74^.

#### Rabbit wound infection and the administration of phage and vancomycin treatment

Wounds of rabbits received 400 μl (10^4^ CFU/mL) of MRSA and 400 μL (10^6^ PFU/mL) of bacteriophage, i.e., the multiplicity of infection (MOI) is 100, which is the ratio of viral plaque-forming units (PFU) to bacterial (CFU). 400 µl containing 5 µg vancomycin was also applied according to Table 3.

**Table 3:** Semi-quantitative scale for the histological parameters.

Group	Ulceration	Vascular proliferation	Inflammation	fibrosis	IL-6	TNF-α
Infected wound (not treated)	+++	+	+++	Moderate	+++	+++
Infected wound treated with vancomycin	++	++	++	Moderate	+++	+++
Infected wound treated with phage	+++	+	+++	Marked	++	++
Infected wound treated with phage and vancomycin	+	++	+	Moderate	+	+
Prevention Group	+++	+	+++	Moderate	++	+++

IL-6: Interleukin 6, TNF-α: Tumor necrosis factor-alpha.

#### Analysis of wound contraction percentage

The wound was photographed using a digital camera. The following equation was used to compute the % wound closure rate:

Wound closure (%) = [1 − (Wound area on given day∕ Wound area on day 0)] × 100.

The percentage change was calculated for each rabbit using 100% as the wound area of each rabbit at the start of the experiment^66^.

For image analysis of wounds, the scale bar of wound healing size and average wound width was performed using imageJ® software^75^.

### Histopathological analysis and immunohistochemistry (IHC) of wound healing

#### Histological examination

On the 17^th^ day, the rabbits were sacrificed, followed by the removal of the tissue from the wound and its surrounding healthy skin to assess the skin wound healing. For histopathological investigations, the tissue was fixed in 10% formaldehyde and embedded in paraffin blocks. The skin sections were stained with hematoxylin and eosin (H&E) and examined under a light microscope (Leica, Germany). A histological examination of the whole wound area was done. The mean value of the percentage of ulceration, vascular proliferation, and inflammation. Tissue fibrosis was quantified using ImageJ software, v1.53 (Maryland, USA) after staining the section with Picro-Sirius red stain^76^.

Interpretation of quantitative assessment of tissue fibrosis was done as follows:

The percentage area of compact collagen in each section was calculated by using the ImageJ® software. Compact collagen < 25% of the measured tissue area was considered as (mild) fibrosis; area > 25 and less than 50% was considered moderate fibrosis, while compact collagen area ≥ 50 was considered marked fibrosis.

#### IHC staining and interpretation

IHC of interleukin-6 (IL‑6), and tumor necrosis factor-alpha (TNF-α) cytokines was done on paraffin-embedded tissue sections to predict the wound progression. The procedure involved the binding of a primary antibody to the antigen of interest and the detection of the bound antibody by avidin–biotin peroxidase plus 3,3′-diaminobenzidine tetrahydrochloride (DAB) (Universal detection kit ‘Envision’ from DAKO, Denmark). A rabbit monoclonal antibody against TNF-α protein was used as the primary antibody (Ab-1Golden, Lab Vision Clone 2D2, Santa Cruz Biotechnology Inc., Santa Cruz, California, USA. Cat.#MS-52B83). A rabbit monoclonal antibody against IL-6 was used as the primary antibody (Ab-1, CloneQBEnd/10, Lab Vision Corporation Laboratories, CA 6521, USA, Cat. #MS-363-R7). Negative control sections for TNF-α protein and IL-6 were treated with non-specific immunoglobulin instead of a specific antibody. The reactivity degree was classified on a scale as weakly positive, moderately positive, and strongly positive. These measurements were obtained by the ImageJ commercial software under magnification × 400 as the area% of positive immunoreactions for TNF-α protein and IL-6^77^.

### Molecular assessment of Wound Healing biomarkers

#### Extraction of RNA and quantitative real-time PCR (qPCR)

RNA extraction was done from the tissue of the wound and blood samples using GENEzol™ TriRNA Pure Kit, Catalogue number: GZXD050. cDNA was performed using a Viva cDNA synthesis kit (Product No. cDSK01-050). The SYBR Green PCR Master mix kit (Qiagen, USA) was used for the PCR reactions. The relative expression of the target genes (collagen, MMP1, PDGF, FGF2, IL-1, IL-6, and TNF‑α) was calculated against the reference gene, GAPDH. The sequences of primers are shown in Table 4. The relative expression was determined using the comparative cycle threshold (Ct) (2 − ) method^78,79^.

**Table 4:** The primer sequence and accession number for quantitative real-time PCR.

Target gene	Accession number	Primer sequence
GAPDH	NG_027767	Forward 5-CTGAACGGGAAACTCACTGG-3 Reverse5-TCACCACCTTCTTGATGTCG-3
TNF-α	NM_001082263	Forward5-GTCTTCCTCTCTCACGCACC-3 Reverse5-TGGGCTAGAGGCTTGTCACT-3
Collagen I	XM_017348831	Forward5-AAGGACACAGAGGTTTCAGTGGTT-3 Reverse5-GCAGCACCAGTAGCACCATCGTTT-3
MMP1	NM_001171139	Forward5-GCAGAATGAGCTACCAGGATAC-3 Reverse5-CAGAAACAGCAGCGTCAATATG-3
IL-1β	NM_001082201	Forward5-CCACAGTGGCAATGAAAATG-3 Reverse5-AGAAAGTTCTCAGGCCGTCA-3
IL-6	NM_001082064.2	Forward5-GAACAGAAAGGAGGCACTGG-3 Reverse5-CTCCTGAACTTGGCCTGAAG-3
PDGF	XM_002710305	Forward5-CTCGTCCTTGCTCATGTCCATGTA-3 Reverse5-GCACACCTTCCTGCAGCAGCACTCCG-3
FGF2	XM_002717238.3	Forward5-GCTGTACTGCAAAAACGGGG-3 Reverse5-TGATGTGTGGGTCGCTCTTC-3

GAPDH: glyceraldehyde-3-phosphate dehydrogenase, TNF-α:tumor necrosis factor alpha, MMP1:matrix metallopeptidase 1, IL-1β:interleukin 1, IL-6:interleukin 6, PDGF:platelet-derived growth factor, and FGF2:fibroblast growth factor 2.

### Statistical analysis

All experiments were conducted in technical triplicates. Rabbit groups were conducted as independent biological triplicates. All the statistical details of experiments can be found in the figure legends, figures, and results. The results were illustrated as mean ± standard deviation (SD). In this study, GraphPad Prism 8 software was used to generate graphs and perform all statistical analyses. Both Student’s *t*-test (two-tailed) and ANOVA test followed by Tukey’s multiple comparisons test were used during the work to evaluate the significance *p* < 0.05.

## Supplementary Information

Supplementary Information.

## Data Availability

All data supporting the findings of this study are available within the manuscript.

## Abbreviations

PAS: Phage-antibiotic synergy
MIC: Minimum inhibitory concentrations
MitC: Mitomycin C
EOP: Efficiency of plating
FICI: Fractional inhibitory concentration index
IHC: Immunohistochemistry
H&E: Hematoxylin and eosin
SD: Standard deviation
VRSA: Vancomycin-resistant Staphylococcus aureus
TNF-α: Tumor necrosis factor-alpha
MMP1: Matrix metalloproteinase 1
PDGF: Platelet-derived growth factor
FGF-2: Fibroblast growth factor-2
VRSA: Vancomycin-resistant Staphylococcus aureus
SICU: Surgical intensive care unit
CA-MRSA: Community-acquired MRSA
ARRIVE: Animals in research: reporting in vivo experiments
