# Supplementary material for: Phage-antibiotic synergistic effect for treating cutaneous wounds infections caused by MRSA and the assessment of wound healing biomarkers in a rabbit model
Source: Sci Rep. 2026 Feb 12;16:6337. doi: 10.1038/s41598-025-34474-6 (PMC12905222; doi:10.1038/s41598-025-34474-6)
Supplement: Supplementary file 1 — Supplementary Information. [file 41598_2025_34474_MOESM1_ESM.docx]

**Table S1.** Investigation of the susceptibility of MRSA isolates to prophage

| **Bacterial isolates** | **Phage** | **Bacterial isolates** | **Phage** |
| --- | --- | --- | --- |
| 1 | Clear | 11 | Clear |
| 2 | Not clear | 12 | Clear |
| 3 | Clear | 13 | Clear |
| 4 | Clear | 14 | Not clear |
| 5 | Not clear | 15 | Clear |
| 6 | Not clear | 16 | Not clear |
| 7 | Clear | 17 | Clear |
| 8 | Clear | 18 | Not clear |
| 9 | Clear | 19 | Not clear |
| 10 | Clear | 20 | Not clear |

The spot test was done with undiluted phage stock (approximately 1 × 10^10^ PFU). Results were evaluated as “Not clear” when no inhibition zone was observed; “Clear” when clear spots were detected.

**Table S2.** Wound area for different groups

|  | Area (cm^2^) | | | | | |
| --- | --- | --- | --- | --- | --- | --- |
| Group | | Day 0 | Day 3 | Day 7 | Day 10 | Day 17 |
| Challenged with MRSA bacteria only (no treatment, control group) | | 3.14 | 2.54 | 2.01 | 1.54 | 1.13 |
| Challenged with MRSA bacteria then after 2 hr vancomycin was applied | | 3.14 | 1.77 | 1.33 | 0.95 | 0.64 |
| Challenged with MRSA bacteria then after 2 hr phage was applied | | 3.14 | 1.33 | 0.95 | 0.64 | 0.38 |
| Challenged with MRSA bacteria then after 2 hr phage & vancomycin were applied | | 3.14 | 0.95 | 0.64 | 0.38 | 0.2 |
| Received phage then after 2 hr were challenged with MRSA (prevention group) | | 3.14 | 2.01 | 1.54 | 1.13 | 0.79 |

**Table S3.** Wound closure percentages for different groups

|  | Percentage of wound closure | | | | | |
| --- | --- | --- | --- | --- | --- | --- |
| Group | | Day 0 | Day 3 | Day 7 | Day 10 | Day 17 |
| Challenged with MRSA bacteria only (no treatment, control group) | | 0 | 19% | 35.98% | 50.96% | 64.01% |
| Challenged with MRSA bacteria then after 2 hr vancomycin was applied | | 0 | 43.6% | 57.6% | 69.7% | 79.61% |
| Challenged with MRSA bacteria then after 2 hr phage was applied | | 0 | 57.6% | 69.7% | 79.61% | 87.89% |
| Challenged with MRSA bacteria then after 2 hr phage & vancomycin were applied | | 0 | 69.7% | 79.61% | 87.89% | 93.63% |
| Received phage then after 2 hr were challenged with MRSA (prevention group) | | 0 | 35.98% | 50.96% | 64.01% | 74.84% |

**
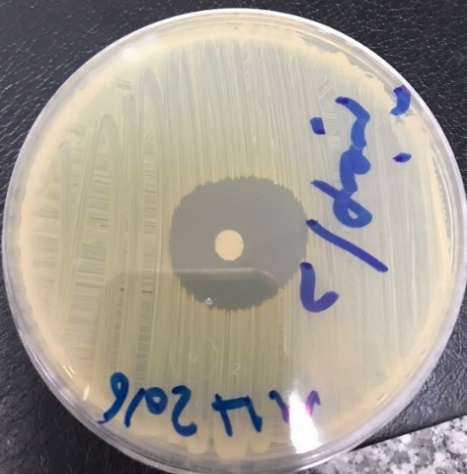
**
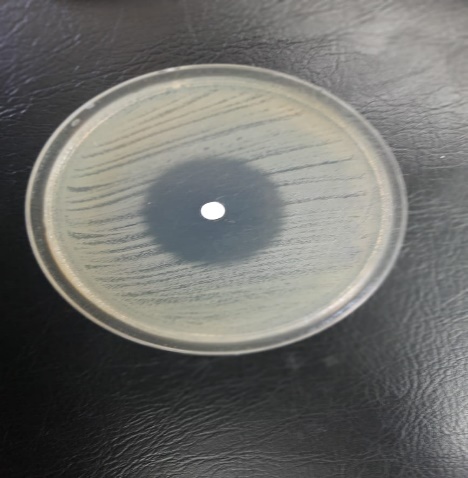

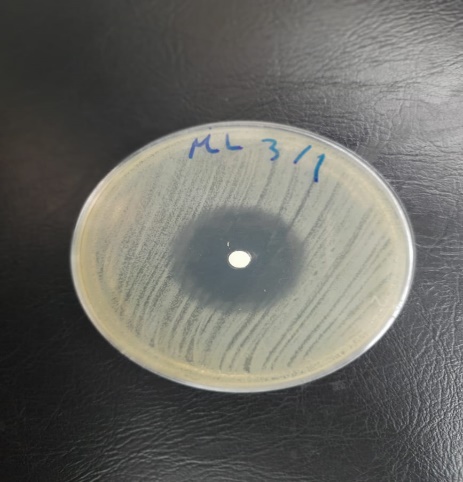


**Fig S1.** The vancomycin sensitivity disk diffusion method shows a zone of inhibition (ZOI), and that MRSA isolates are susceptible to vancomycin.A
